# Supplementary figures and images for: Hippo pathway-mediated YAP1/TAZ inhibition is essential for proper pancreatic endocrine specification and differentiation
Source: eLife. 2024 Jul 25;13:e84532. doi: 10.7554/eLife.84532 (PMC11272159; doi:10.7554/eLife.84532)

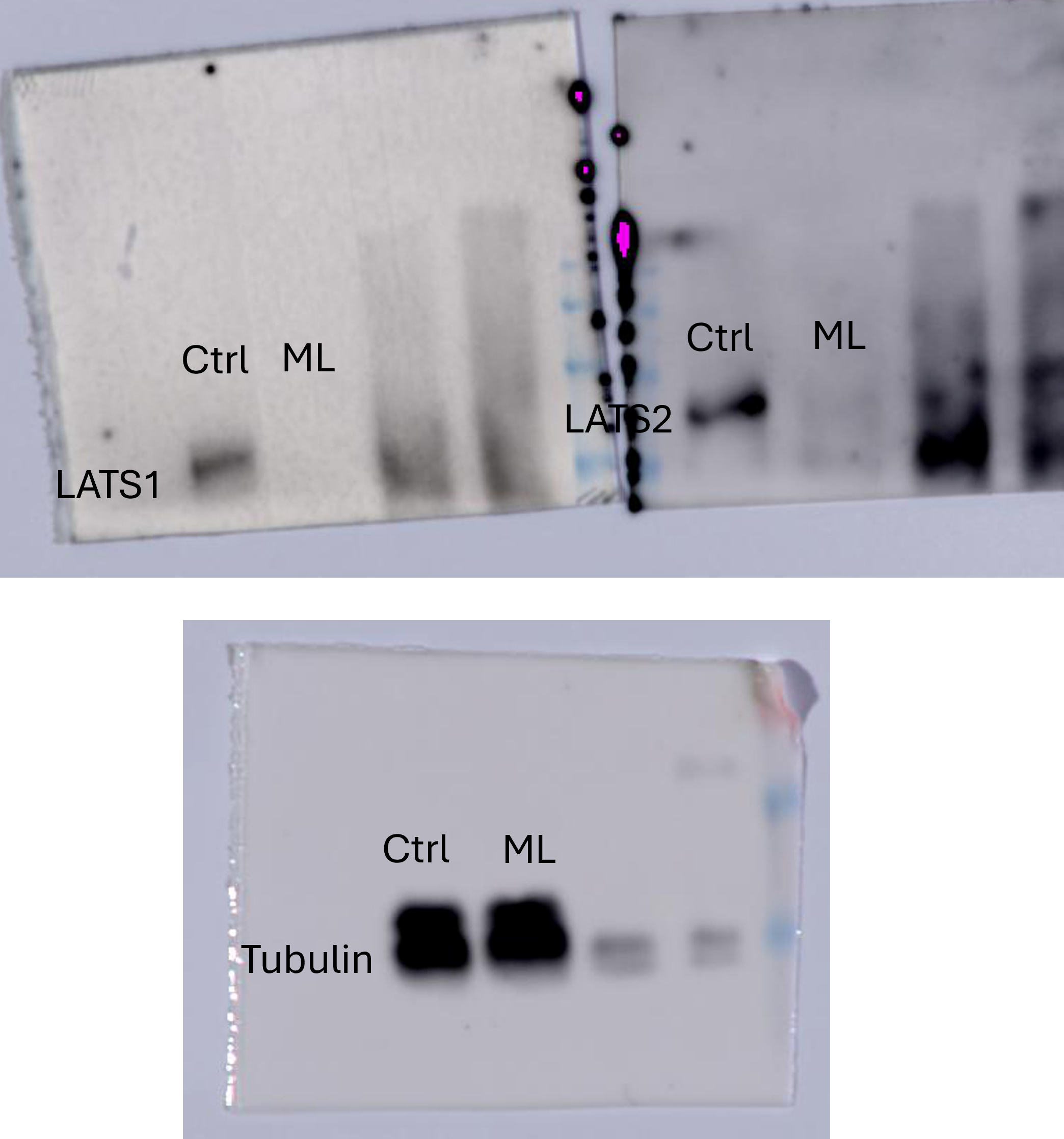

Supplement: Figure 8—source data 1. [file elife-84532-fig8-data1.zip › Figure 8-source data 1/blots with label.png]

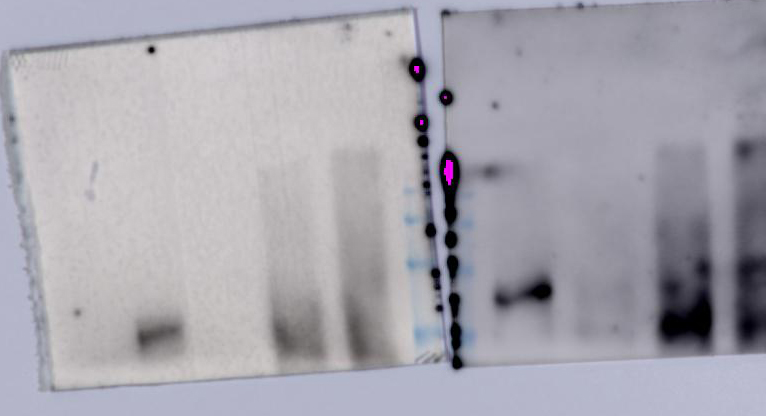

Supplement: Figure 8—source data 1. [file elife-84532-fig8-data1.zip › Figure 8-source data 1/original raw blots for LATS1 LATS2 .jpg]

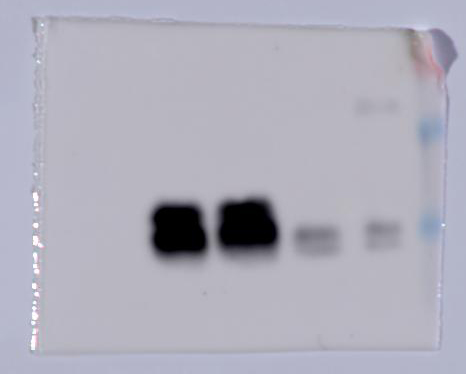

Supplement: Figure 8—source data 1. [file elife-84532-fig8-data1.zip › Figure 8-source data 1/original raw blots for TUBULIN.jpg]
